# Bronchoesophageal Fistula due to Esophageal Tuberculosis

**DOI:** 10.1155/2019/6537437

**Published:** 2019-03-21

**Authors:** Pathik Desai, Prachi Mayenkar, Thomas F. Northrup, Vijaya Mallela

**Affiliations:** ^1^Department of Family Medicine, University of Texas Health Science Center, Houston, TX, USA; ^2^Department of Internal Medicine, University of Texas Health Science Center, Houston, TX, USA

## Abstract

This is a case report regarding a patient who presented with 6 months of dysphagia and subsequent 40-pound weight loss. The patient underwent imaging, suggestive of pulmonary TB. Further workup of his dysphagia with esophagogastroduodenoscopy and bronchoscopy revealed two bronchoesophageal fistulas. Tuberculosis is an important differential diagnosis of prolonged dysphagia in immunocompetent patients.

## 1. Introduction

Reports of esophageal manifestations of tuberculosis are rare and can be a secondary manifestation of pulmonary tuberculosis (TB), or in even rarer cases, primary esophageal tuberculosis [1]. Esophageal TB is commonly noted in developing countries and presents with prolonged dysphagia that affects the middle third of the esophagus [1]. In developed countries, esophageal TB may be initially misdiagnosed as esophageal malignancy [2]. However, prompt diagnosis and treatment with antituberculous therapy is imperative as any delay in treatment when esophageal manifestations are present, may lead to complications such as ulcerations, esophageal stenosis, and tracheoesophageal fistulas, which may require surgical management [1, 3]. The following report describes a case in which *Mycobacterium tuberculosis* (MTB) infection exhibited esophageal involvement in an immunocompetent patient.

## 2. Case Presentation

The patient was a 46-year-old Hispanic male who presented with a six-month history of productive cough and pleuritic chest pain associated with intermittent episodes of fevers and chills. Approximately three months into his symptoms, the patient began to experience dysphagia and subsequent decreased appetite. His dysphagia progressed from solids to liquids. He denied having trouble breathing. The patient reported an unintentional 40-pound weight loss over the three-month period following the onset of his dysphagia. His vital signs were within normal limits, and the patient appeared his stated age. The physical exam was remarkable for cachexia.

**Figure 1:**
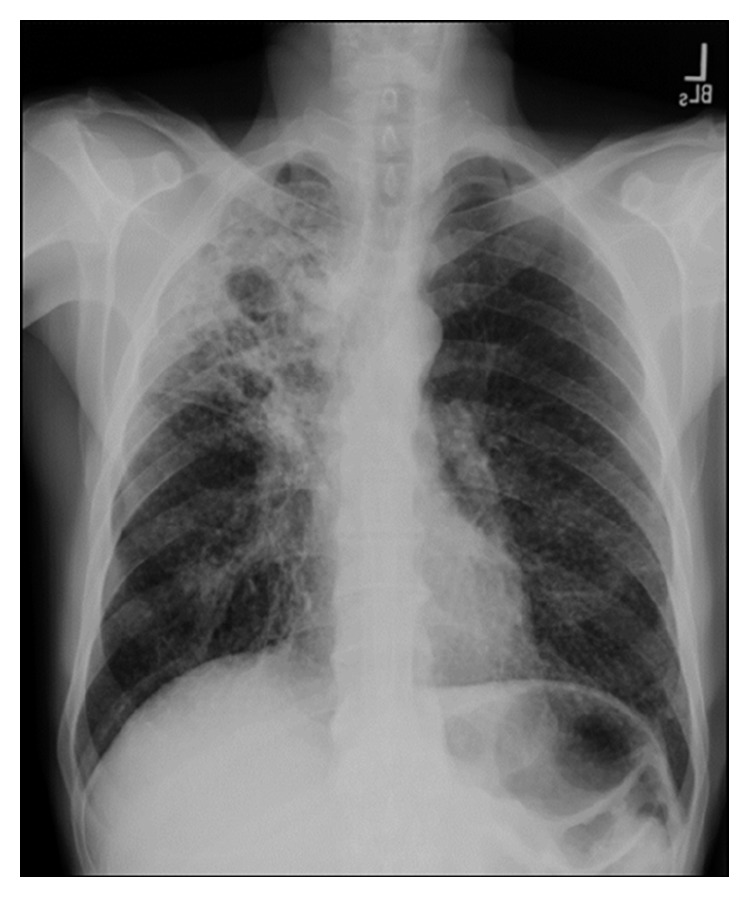
Chest X-ray showing cavitary opacity in the right upper lung field.

**Figure 2:**
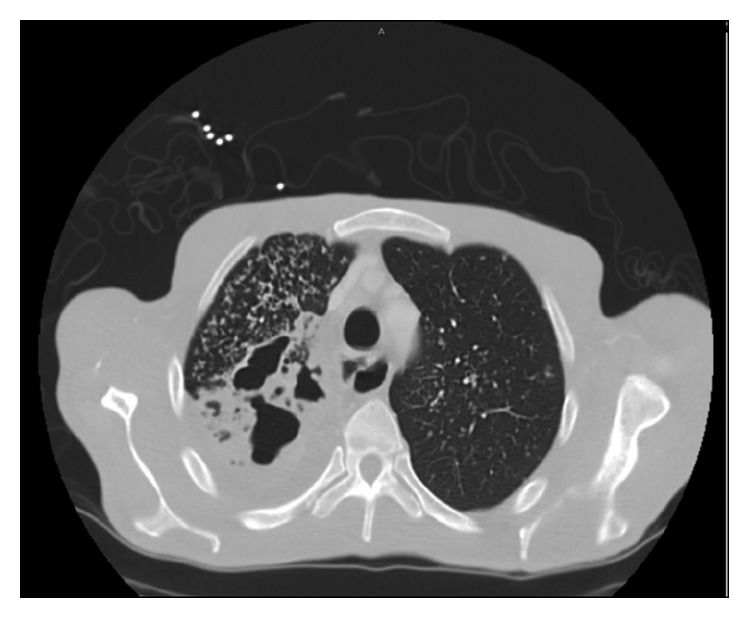
Contrast-enhanced CT scan. Axial section (neck) showing airspace consolidations with diffuse tree in bud opacities in the right lung apex and to a less extent, in the left lung apex. In addition, there are multiple areas of cavities within the right lung apex.

Initial lab work was unremarkable. Initial chest X-ray revealed a prominent right upper lobe cavitary lesion (Figure 1). Further workup was obtained with a CT chest, which showed “tree-in-bud” opacities in bilateral lung fields, along with “thick walled” cysts of the right upper lobe (Figure 2). This was reported to be suggestive of TB versus fungal infection. Additionally, the imaging revealed mediastinal lymphadenopathy. Sputum acid-fast bacilli (AFBs) were collected, and the patient started treatment with rifampin, isoniazid, pyrazinamide, and ethambutol (commonly referred to as RIPE therapy), prior to sputum acid-fast bacilli cultures returning positive [4].

**Figure 3:**
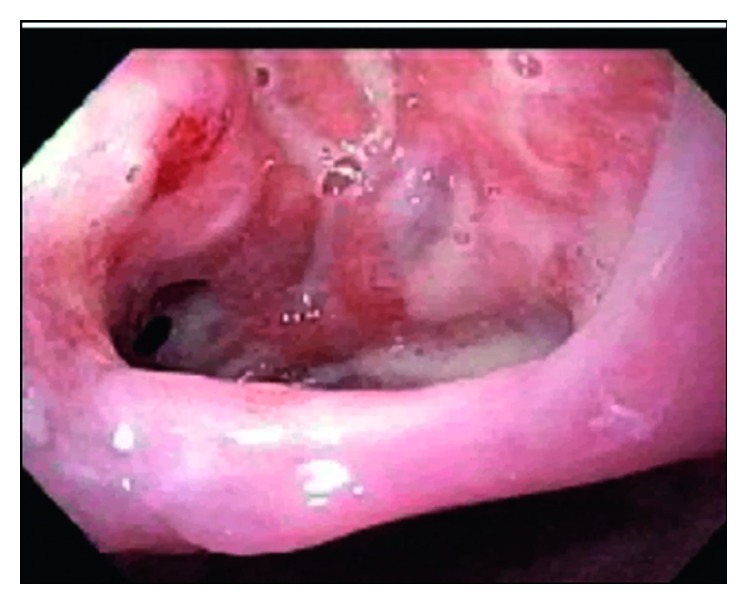
Esophagogastroduodenoscopy (EGD). The upper esophagus at 25 cm showed a 2.5 cm defect with a visible fistulous track to the trachea.

**Figure 4:**
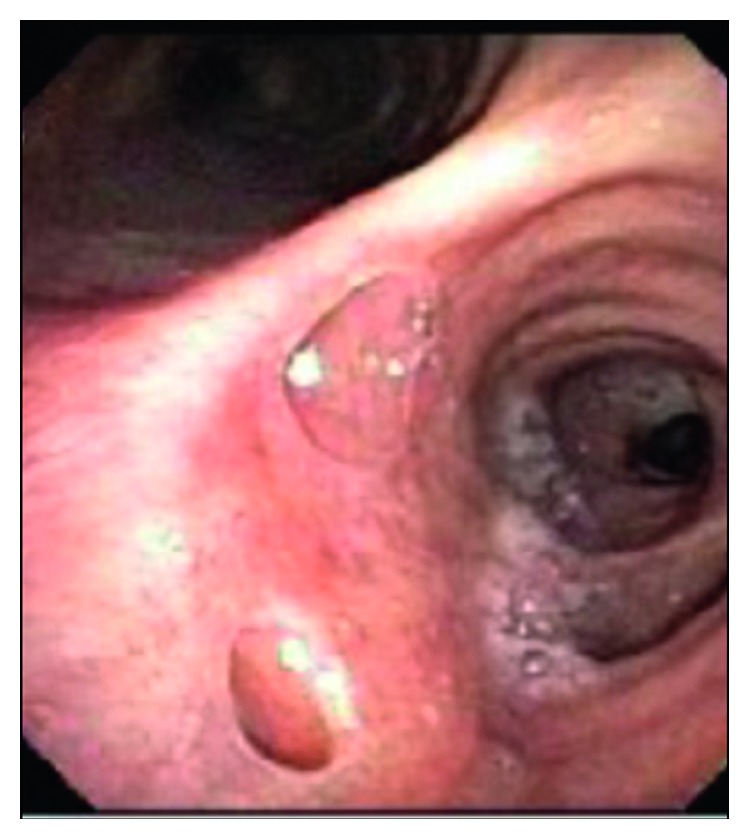
Bronchoscopy. Bronchoesophageal fistulas with openings seen in the right mainstem bronchus on the posterior aspect right below the carina at around 6'o clock position.

**Figure 5:**
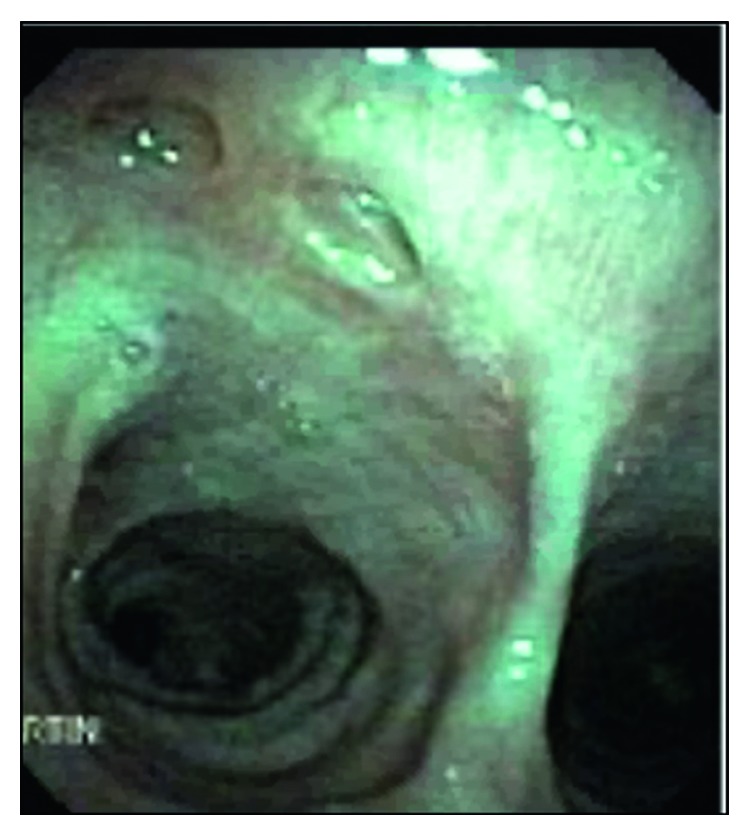
Bronchoscopy (posttreatment). There are two healed fistulas in the right posterior mainstem (posterior to carina), measuring approximately 0.5 cm each.

To determine the etiology of the dysphagia, the patient underwent a swallow evaluation which revealed combined oral and pharyngeal dysphagia. Follow-up studies with an esophagogastroduodenoscopy (Figure 3) and bronchoscopy (Figure 4) revealed bronchoesophageal fistulas, which were presumed to be a result of MTB infection. Given his inability to tolerate oral nutrition, a percutaneous endoscopic gastrostomy (PEG) tube was placed for nutritional purposes. Once he was able to tolerate PEG tube feeds, he was discharged home on RIPE therapy and given a follow-up appointment in an infectious disease clinic. At a three-month follow-up appointment on RIPE therapy, the patient was able to tolerate oral nutrition. He was reevaluated with a bronchoscopy (Figure 5), which showed interval healing of the bronchoesophageal fistulas. Since the patient was tolerating a regular diet, the PEG tube was scheduled to be removed three months after it was placed. The patient followed up at nine months in the clinic. At that time, he reported no symptoms. He completed a nine-month course of RIPE therapy with no further complications.

## 3. Discussion

Esophageal manifestations of MTB are extremely rare, and there are only a few documented reports. In this case, the patient was experiencing six months of dysphagia. He likely experienced complications of esophageal TB because his symptoms were not timely addressed, due to his delay in seeking medical treatment. In cases with only esophageal involvement, primary symptoms are reported to be prolonged dysphagia, retrosternal pain, and weight loss [1, 2, 5–7]. Most patients, however, with esophageal involvement of TB will also have chest imaging findings consistent with pulmonary TB. The most common manifestation of esophageal TB is a tracheoesophageal fistula and mediastinal lymphadenopathy [8]—both of which were seen in this patient. It has been proposed that the mechanism of esophageal involvement of MTB is through the ingestion of sputum that is infected or by the hematogenous spread from pulmonary TB [8, 9]. Individuals with esophageal tuberculosis involvement may be effectively treated using RIPE therapy for a duration of six to nine months [4, 10]; further prolonged treatment is not necessary, even with the presence of anatomic anomalies such as tracheoesophageal fistula, ulcerations, and esophageal stenosis, unless the patient is infected with drug-resistant organisms [2, 9]. Surgery may be necessary for cases of tracheoesophageal fistula [10]; however, in this case, the patient experienced resolution of the fistula after only a few months of antituberculous therapy. The incidence of healing may vary depending on severity of fistula and time to accurate diagnosis and treatment.

## 4. Conclusions/Recommendations

Although rare, esophageal TB is an important rule-out in patients with persistent dysphagia. The infection is primarily seen in developing countries and is important to keep in mind in developed nations, particularly for immigrant populations and immunocompromised patients [2, 3, 5, 8]. In this case, the RIPE therapy was curative; however, this patient's esophageal symptoms could have worsened had the patient gone untreated. Any patient who presents to a clinical setting with a prolonged history of dysphagia and any anatomical anomaly of the esophagus (i.e., stricture, fistula, and ulcerations) should undergo further workup for MTB infection, along with keeping other disease states in the diagnostic differential.
